# Treatment of Dystonia Using Trihexyphenidyl in Costello Syndrome

**DOI:** 10.3390/brainsci10070450

**Published:** 2020-07-14

**Authors:** Domenico M. Romeo, Alessandro Specchia, Alfonso Fasano, Chiara Leoni, Roberta Onesimo, Claudia Brogna, Stefania Veltri, Giuseppe Zampino

**Affiliations:** 1 Pediatric Neurology Unit, Fondazione Policlinico A. Gemelli, IRCCS, 00168 Rome, Italy; 2 Pediatric Neurology Unit, Università Cattolica del Sacro Cuore, 00168 Rome, Italy; claudiabrogna@yahoo.it (C.B.); stefaniaveltri@hotmail.it (S.V.); 3 Physical and Rehabilitation Unit, Fondazione Policlinico A. Gemelli, IRCC, 00168 Rome, Italy; alespecchiamd@gmail.com; 4 Edmond J. Safra Program in Parkinson’s Disease and the Morton and Gloria Shulman Movement Disorders Centre and the, Toronto Western Hospital, UHN, Toronto, ON M5T 2S8, Canada; alfonso.fasano@gmail.com; 5 Division of Neurology, Department of Medicine, University of Toronto, Toronto, Canada, Krembil Brain Institute, Toronto, ON M5T 1M8, Canada; 6 Rare Diseases and Birth Defects Unit, Fondazione Policlinico A. Gemelli, IRCCS, 00168 Rome, Italy; chiara.leoni@policlinicogemelli.it (C.L.); roberta.onesimo@policlinicogemelli.it (R.O.); giuseppe.zampino@unicatt.it (G.Z.)

**Keywords:** Costello syndrome, dystonia, trihexyphenidyl, personalized medicine

## Abstract

Costello syndrome (CS), a rare syndrome with multisystemic involvement inherited as a dominant trait, is characterized by developmental delay, coarse facial appearance, cardiac defects including hypertrophic cardiomyopathy, skin abnormalities, brain complications, and a predisposition to certain malignancies. The musculoskeletal system is particularly affected in CS, with peculiar orthopedic anomalies that impact posture and gait. Dystonia has been recently documented to contribute to abnormal postures and musculoskeletal anomalies characterizing CS, suggesting the possible use of pharmacological treatments to treat these complications. We report the case of a child affected by CS displaying a particularly severe musculoskeletal involvement with dystonic posture especially in the arms and legs. The Movement Disorder-Childhood Rating Scale (MD-CRS) and a gait analysis were used to assess clinical patterns of hyperkinetic movement disorder and dystonia. The child was further treated with trihexyphenidyl for six months with a final dosage of 14 mg. MD-CRS and gait analysis assessments provided evidence for a significant improvement of posture and the related musculoskeletal problems with no side effects. Our preliminary study report provides first evidence that pharmacological anti-dystonia treatment significantly improves movement and posture disorders in patients with CS. Further studies enrolling larger cohorts of patients should be performed to validate these preliminary observations.

## 1. Introduction

Costello syndrome (CS) is a rare syndrome with multisystemic involvement inherited as a dominant trait and caused by a restricted spectrum of germline missense mutations in the *HRAS* proto-oncogene [1]. CS is clinically related to other disorders affecting development and growth that are collectively termed RASopathies, which share aberrant signaling through *HRAS* and downstream signaling cascades as the molecular mechanism underlying pathogenesis [2,3]. CS is characterized by prenatal overgrowth, which is followed by postnatal feeding difficulties, severe failure to thrive, and reduced growth. Other features include developmental delay, coarse facial appearance, cardiac defects including hypertrophic cardiomyopathy, skin abnormalities, brain malformations (Chiari I malformation, ventriculomegaly), and a predisposition to certain malignancies. About 80% of affected cases reported to date, harbor the pathogenic c.34G>A; p.Gly12Ser variant in the *HRAS* gene [4]. The musculoskeletal system is particularly affected in CS, influencing posture and gait [5,6], and affecting everyday life [7]. Major orthopedic manifestations include hypotonia, ligamentous laxity, a distinctive hand appearance (short, broad, hyperextensible digits with ulnar deviation), decreased shoulder and elbow range of motion, tight Achilles tendons and foot abnormalities, scoliosis, kyphosis, lordosis, and dysfunctional gait. Some of these features, such as ulnar deviation and flexion of the wrists, have a high prevalence and are not reported in other RASopathies, occur early during fetal development, and can be disabling when particularly severe. CS patients frequently require the use of orthosis and postural/mobility devices to improve posture and facilitate the acquisition of neurodevelopmental milestones. While the factors contributing to these complications still need to be understood more precisely, these observations document that orthopedic management is essential for proper care of subjects with CS.

In a recent study based on six patients with CS aged between 7 and 27 years, it was suggested that dystonia contributes to the abnormal posture characterizing CS [8]. Specifically, although the presence of osteoarticular anomalies exists, abnormal postures could be passively reduced and spontaneously fluctuated over seconds. The authors also noticed signs of generalized dystonia and dystonic posturing of hands and feet, such as internal rotation of feet, big toe extension, dystonic thumb extension, reducible wrist flexion, and a variety of chorea-like and dystonic jerky movements of the hands; they also suggested that dystonia could be considered a key factor contributing to the abnormal postures in CS patients, previously misinterpreted as orthopedic manifestations of unknown origin.

These considerations were predicted to have direct impact in the clinical practice as they suggest the possible use of physiotherapy and pharmacological anti-dystonia treatment for these complications. Moreover, early interventions for dystonic symptoms could also significantly contribute to improve the motor organization and psychomotor development of these patients. 

## 2. Case Report

We report the case of a child affected by CS displaying typical clinical characteristics of the syndrome and a particularly severe musculoskeletal involvement. This eight-year-old boy was submitted to a neurological evaluation for abnormal posture. The child was delivered to healthy and non-consanguineous parents at 35 weeks of gestational age with cesarean section for fetal asphyxia (Apgar score 3, 5), and treated in the neonatal intensive care unit. Weight at birth was 3.650 g. Due to the peculiar craniofacial features, pigmented skin, hypertrophic cardiomyopathy, prenatal overgrowth and postnatal feeding difficulties resulting in severe failure to thrive, a clinical diagnosis of CS was made, which was confirmed by molecular analysis revealing the recurrent c.34G>A; change (p.Gly12Ser) de novo variant in the *HRAS* gene (NM_001130442.2; ClinVar ID: 12602). Written informed patient consent was obtained and a Patient’s Consent to Publication was reported in a separate file.

Independent walking was acquired at the age of three years, after Achilles tendons resection; global developmental delay was delineated with particular impairment of gross motor and fine motor abilities. He performed speech and physiotherapy treatment (two days/week) from three years of age, and ankle-foot-orthoses were used without significant improvements. Cranial MRI showed small posterior fossa with Chiari I malformation, and ventriculomegaly of a slight degree, which represent typical findings in children with CS [9]. At the age of eight years, neurological assessment documented normal muscle tone at rest with the presence of dystonia during movements and in maintaining postures. Deep tendon reflexes were normal. A dystonic posture was observed as internal rotation of feet, big toe extension, and dystonic thumb extension. A distinctive hand posture, characterized by a reducible wrist flexion, and ulnar deviations of fingers and dystonic jerky movements of the hands were also reported. Based on these findings, the Movement Disorder-Childhood Rating Scale (MD-CRS) [10] and a gait analysis were performed. MD-CRS assessment is specifically validated for pediatric patients and provides a general assessment (Index I) with 15 items and four areas included (motor function, oral/verbal function, self-care, and attention/alertness), a movement-disorder severity (Index II, 7 item), and a global index, to evaluate the influence of various movement disorders on daily living activities. Each index can range from 0 to 1 and it is divided into five classes, according to the severity of movement disorder. Class 1 included a 0–0.2 index (healthy), class 2 a 0.2–0.4 index (mildly affected), class 3 a 0.4–0.6 index (moderately affected), class 4 a 0.6–0.8 index (severely affected), and class 5 a 0.8–1 index. The MD-CRS has successfully been used in children with various movement disorders due to different etiologies, demonstrating to be a sensitive tool to detect different profiles of drug response, quantifying the rate of changes in these patients [11,12]. By providing a general assessment and a movement-disorder severity scoring, our child initially reported a final Global Index (Class II, mildly affected).

For the gait analysis, spatiotemporal parameters (step and stride lengths, cadence, and walking velocity) and lower extremity kinematics at the pelvis, hip, knee, and ankle were collected [13]. Barefoot walking trials at a self-selected speed were collected and the mean of three trials was calculated and used in this analysis.

Based on the collected evidence indicating occurrence of dystonia, treatment with trihexyphenidyl was initiated, using a starting dose of 0.5 mg/kg/day, then gradually increased to the final dosage of 7 mg twice daily. After a period of six months, the same outcome measures were repeated. Although the child reported the same class of severity of movement disorder (Table 1), an improvement was documented for specific tasks of the MD-CRS (Index II) in upper limb (reaching, grasping, and graphism). Gait analysis documented an improvement in most of the spatiotemporal and kinematic parameters (Table 2, Figure S1a,b): increased range of motion (ROM) of the hip, knee, and ankle both in stance and swing phases; enhanced pelvis stability (reduced tilt and obliquity ROM); and increased stride length and mean walking and swing speed with step width reduction (Video S1). No side effects were reported during the treatment and follow-up period. The child continued to perform the same rehabilitation protocol during the treatment with trihexyphenidyl.

## 3. Discussion

CS is a rare genetic disease caused by a germline mutation in the HRAS proto-oncogene. Previous work documented the occurrence of dystonia as one of the most frequent neurological features in CS, contributing to the abnormal postures previously described in these patients [6]. In fact, in these patients, although there are osteoarticular abnormalities [5,6], some abnormal postures can be passively reduced and may spontaneously fluctuate over seconds. Furthermore, genotype/phenotype correlation showed how, within patients affected by CS, those carrying the Gly12Ser variant, as our patient, had a higher prevalence of musculoskeletal abnormalities [4]. Based on these observations, we hypothesized that currently available pharmacologic approaches directed to treat dystonia could be effective in improving symptoms and the quality of life of these patients; therefore, trihexyphenidyl was used to treat abnormal posture and movements in a child with CS, exhibiting a particularly severe dystonia. Clinical assessment of the child after treatment documented a significant improvement in a six months follow-up period, in movement-disorder severity and in the global index of the MD-CRS, a scale specifically developed for movement disorders; mainly it was observed in global motor improvements, especially in specific tasks of daily living activities such as reaching, grasping, and graphism. Gait analysis also documented an improvement in most of the spatiotemporal and kinematic parameters, mainly in pelvis stability, hip and knee flexion-extension, stride length, and walking speed. No side effects were reported during the treatment. 

Trihexyphenidyl is one of the most used drugs in children to improve dystonia [14], thanks to its effects on tone reduction and an overall functional improvement. The specific anti-dystonia mechanism of trihexyphenidyl is not well understood but it is probably related to its antimuscarinic effect [15]. In knock-in mice with primary dystonia (Dyt1 KI), the muscarinic anti-cholinergic receptor (mAChR) antagonism is specifically required to offset plasticity deficits [15].

Recent evidence suggests that dystonia may result from the disruption of a motor network involving the basal ganglia and cerebellum, which leads to enhanced motor cortex excitability, a feature that has also been observed in CS [16]. Consistently, the *HRAS* signaling network, which is altered in CS patients, plays a key role in synaptic plasticity and in the functional and structural organization of networks in the cerebral cortex [17,18]. Of note, in CS patients, megalocephaly and cerebellar enlargement, probably caused by abnormal genesis of neurons and/or glia, represent relatively common features [17]. The coexistence of cerebellar abnormalities and altered sensorimotor plasticity could suggest the dystonic quality in the abnormal postures in CS [8,17,18].

The good response to trihexyphenidyl, on one hand, confirms the presence of dystonia in our children with CS, as recently reported, opening good scenarios for possible future clinical trials [6,19]; on the other hand, our preliminary results have therapeutic implications demonstrating specific impact on functional activities, as detected by the MD-CRS and gait analysis. As the child had already reported some musculoskeletal deformities before the beginning of the treatment, the effect of the trihexyphenidyl could have a minor impact in the global class of severity of movement disorder.

## 4. Conclusions

In conclusion, the present case report provides first evidence that pharmacological anti-dystonia treatment significantly improves movement and posture disorders in patients with CS. However, it is not possible at the moment to reach a definitive conclusion on the use of the anti-dystonic treatment in children with Costello syndrome. Further studies enrolling larger cohorts of patients should be performed to validate these preliminary observations; the clinical relevance of orthopedic complications in CS encourages pharmacological trials directed at an early treatment of dystonic symptoms in CS patients to prevent fixed deformity. As the musculoskeletal phenotype of CS evolves along with the patient’s life, it is possible that at older ages, the disabling osteoarticular abnormalities could be no longer modifiable, and surgery could be considered as the only possible approach.

Therefore, we recommend that these patients undergo a more comprehensive clinical neurological assessment as soon as possible, also devoted to identifying the occurrence and treatment of dystonia.

## Figures and Tables

**Table 1:** Movement Disorder-Childhood Rating Scale results.

	Index I	Class I	Index II	Class II	Global Index	Global Class
T0	0.31666667	2	0.321429	2	0.318181818	2
TI	0.31666667	2	0.285714	2	0.306818182	2

**Table 2:** Mean (SD) of selected spatiotemporal and kinematic parameters before (T0) and six months after treatment (T1).

Parameter	T0		T1	
	Right	Left	Right	Left
Walking speed (m/s)	0.63 (0.17)	0.65 (0.16)	1.04 (0.03)	1.07 (0.02)
Cadence (steps/min)	141.5 (16.6)		199.4 (6.9)	
Step length (m)	0.23 (0.04)	0.26 (0.03)	0.31 (0.02)	0.28 (0.01)
Stride length (m)	0.53 (0.08)	0.54 (0.08)	0.63 (0.02)	0.64 (0.02)
Stance (% GC)	62.6 (2.4)	61.2 (1.8)	56.9 (0.4)	58.3 (1.3)
Swing (% GC)	37.4 (2.4)	38.9 (1.8)	43.1 (0.4)	41.7 (1.3)

GC, gait cycle; m, meter; s, second.

## Supplementary Materials

The following are available online at https://www.mdpi.com/2076-3425/10/7/450/s1, Figure S1a,b: Lower extremity kinematic variables pre-treatment and post-treatment. A mean normal curve, the gray area , is shown for comparison. Lower right extremity is reported in dark gray line. Lower left extremity is reported in light gray line. Video S1: The video shows the gait pattern of the child during the gait analysis before and after six months of pharmacological treatment.
